# Design of a randomized controlled trial to assess the comparative effectiveness of a multifaceted intervention to improve adherence to colorectal cancer screening among patients cared for in a community health center

**DOI:** 10.1186/1472-6963-13-153

**Published:** 2013-04-29

**Authors:** David W Baker, Tiffany Brown, David R Buchanan, Jordan Weil, Kenzie A Cameron, Lauren Ranalli, M Rosario Ferreira, Quinn Stephens, Kate Balsley, Shira N Goldman, Michael S Wolf

**Affiliations:** 1 Department of Medicine, Division of General Internal Medicine and Geriatrics, Feinberg School of Medicine, Northwestern University, Chicago, IL, USA; 2 Center for Advancing Equity in Clinical Preventive Services, Feinberg School of Medicine, Northwestern University, Chicago, IL, USA; 3 Erie Family Health Center, Chicago, IL, USA; 4 Department of Medicine, Division of Gastroenterology and Hepatology, Feinberg School of Medicine, Northwestern University, Chicago, IL, USA

## Abstract

**Background:**

Colorectal cancer (CRC) is common and leads to significant morbidity and mortality. Although screening with fecal occult blood testing (FOBT) or endoscopy has been shown to decrease CRC mortality, screening rates remain suboptimal. Screening rates are particularly low for people with low incomes and members of underrepresented minority groups. FOBT should be done annually to detect CRC early and to reduce CRC mortality, but this often does not occur. This paper describes the design of a multifaceted intervention to increase long-term adherence to FOBT among poor, predominantly Latino patients, and the design of a randomized controlled trial (RCT) to test the efficacy of this intervention compared to usual care.

**Methods:**

In this RCT, patients who are due for repeat FOBT are identified in the electronic health record (EHR) and randomized to receive either usual care or a multifaceted intervention. The usual care group includes multiple point-of-care interventions (e.g., standing orders, EHR reminders), performance measurement, and financial incentives to improve CRC screening rates. The intervention augments usual care through mailed CRC screening test kits, low literacy patient education materials, automated phone and text message reminders, in-person follow up calls from a CRC Screening Coordinator, and communication of results to patients along with a reminder card highlighting when the patient is next due for screening. The primary outcome is completion of FOBT within 6 months of becoming due.

**Discussion:**

The main goal of the study is to determine the comparative effectiveness of the intervention compared to usual care. Additionally, we want to assess whether or not it is possible to achieve high rates of adherence to CRC screening with annual FOBT, which is necessary for reducing CRC mortality. The intervention relies on technology that is increasingly widespread and declining in cost, including EHR systems, automated phone and text messaging, and FOBTs for CRC screening. We took this approach to ensure generalizability and allow us to rapidly disseminate the intervention through networks of community health centers (CHCs) if the RCT shows the intervention to be superior to usual care.

**Trial registration:**

ClinicalTrials.gov NCT01453894

## Background

The United States Preventive Services Task Force (USPSTF) recommends colorectal cancer (CRC) screening using fecal occult blood test (FOBT), sigmoidoscopy, or colonoscopy in adults, beginning at age 50 and continuing until age 75 [1]. However, rates of CRC screening remain inadequate [2,3]. In 2006, only 60.8% of adults 50 or older reported recent CRC screening [3]. Screening rates are even lower among Black and Hispanic populations [4-10] and in areas with higher poverty rates [10].

Community health centers (CHCs) frequently use FOBT as their only form of CRC screening due to limited access and high cost of endoscopy. However, FOBT should be done every 1–2 years to optimize detection of polyps and early cancer. Performing the test less often may result in many aggressive cancers being missed until an advanced stage, markedly reducing the health benefits of population screening. Individuals in vulnerable groups often face multiple barriers to annual FOBT screening, including lack of regular source of care, less frequent routine medical visits, frequent changes in residence, and lack of awareness of the need for annual FOBT screening. If FOBT cannot be conducted annually or biennially with high reliability, it may be necessary to expand the use of alternative screening modalities, such as endoscopy (i.e., sigmoidoscopy or colonoscopy), to reduce CRC mortality. However, there is currently inadequate financing and an inadequate number of endoscopists available for CHCs to use endoscopy as a primary screening modality.

Few studies have examined the rate of repeat CRC screening with FOBT [11,12]. To our knowledge, none have been conducted in populations with high prevalence of barriers to screening (e.g., low literacy, varied cultural norms, transportation difficulties). The assumption that FOBT is an effective CRC screening strategy presumes it will be done at least biennially, and cost-effectiveness studies of CRC screening strategies have found that the results are sensitive to the rate of adherence [13]. Therefore, studies are needed to develop and test strategies to increase adherence to FOBT over multiple years. It is important to evaluate a) whether these strategies improve adherence compared to high-quality usual care, and b) whether multifaceted interventions can achieve the consistently high adherence rates year after year that are required to reduce late-stage CRC and CRC mortality.

This paper describes the design of a comparative effectiveness study of an intervention to maximize the number of poor, predominantly Latino patients cared for at a CHC who complete a repeat FOBT within six months of becoming due for CRC screening. We first describe the CHC’s efforts to improve CRC screening rates prior to the start of this study. We then review critical aspects of the study design and intervention, including a) the use of an IRB-approved waiver of informed consent to randomize all eligible patients and achieve a fully representative study population, b) the conceptual framework for the multifaceted intervention, c) the outreach tools developed for each component of the intervention, d) separation of the intervention into two discrete phases to allow assessment of the marginal benefit of outreach by a CRC Screening Coordinator compared to lower-cost outreach strategies, e) the patient educational tools developed to provide feedback to patients with negative FOBTs and to improve successful completion of diagnostic colonoscopy among patients with positive FOBTs, and f) the outcome assessment. Finally, we discuss the significance of the study and some of the potential implications.

### Previous efforts to improve CRC screening rates at Erie Family Health Center

This study is being conducted at Erie Family Health Center (EFHC), a federally-qualified health center network in Chicago, Illinois that serves an overwhelmingly Latino population; 66% are best served in Spanish; 36% are uninsured; and 91% come from households with incomes below the Federal Poverty Line. EFHC uses the General Electric Centricity electronic health record (EHR), which is supported by the Alliance of Chicago Community Health Services. The EHR has clinical reminders for chronic disease management and clinical preventive services and allows EFHC to routinely gather data for quality of care measures.

In December, 2007, 16% of all eligible patients were up to date on CRC screening based on USPSTF guidelines. This rate had been stable over the previous six months and was similar to many other CHCs in Chicago. Between December, 2007 and October, 2009, EFHC implemented several system-level changes designed to improve CRC screening rates. These efforts are best viewed as a single quality improvement initiative with multiple components: a) performance measurement, feedback, and financial incentives, b) implementation of a team-based care approach empowering medical assistants (MAs) to recommend CRC screening, and c) development of strategies to improve access to diagnostic colonoscopy.

#### Performance measurement, feedback, and financial incentives

EFHC uses performance measures to determine provider incentive payments. These measures are chosen every year by the providers themselves to reflect their priorities, rather than being imposed by EFHC’s management team. In 2007, providers chose CRC screening as a performance measure. As a result, adult medicine providers were routinely told what percent of their patient panel was up to date on CRC screening. Along with their individual performance, providers are also shown how their screening rates rank among other adult providers within the organization. Performance on the CRC measure was also used to allocate performance-based incentive bonuses. Each quarter, providers receive a bonus for falling in the top and middle terciles of similar providers in each measure. Along with their individual performance, providers are also shown how their screening rates rank among other adult providers within the organization.

#### Empowerment of medical assistants to offer CRC screening if overdue

EFHC also implemented a standing order that allowed MAs to recommend guaiac card FOBT screening during pre-appointment triage and vital signs measurement to patients who were age 50 or older and not up to date on CRC screening. If the patients agreed to take the FOBT cards home, the MA showed them how to use it and answered basic questions about the test. If patients had questions beyond the MA’s scope of practice, patients were told to discuss this during the provider visit. The provider also had a chance to reinforce the MA’s recommendation to perform the test or to attempt to change a patient’s decision if the patient did not agree to the screening when the MA offered the test.

#### Improved access to diagnostic colonoscopy

Before August, 2009, uninsured EFHC patients had access to diagnostic colonoscopies only through self-pay, charity programs, or care at public hospitals. Each of those modes has limitations. Most uninsured EFHC patients would find the cost of self-financing a colonoscopy not feasible. Charity care applications’ bureaucratic and documentation requirements can be challenging for all patients, especially those with low literacy. Furthermore, public hospitals in the Chicago area tend to have long waitlists for services, especially procedural tests like colonoscopy. These challenges may have caused some providers to be unwilling to use FOBT for CRC screening if they believed a patient could not get a timely diagnostic colonoscopy if the FOBT result was positive.

In August, 2009, EFHC and Northwestern Memorial Hospital implemented an agreement to provide free diagnostic colonoscopies to uninsured patients who met EFHC’s sliding scale fee criteria. Further eligibility requirements included that the patient not be insulin-dependent, be older than age 50 (or 40 if they have a first-degree relative who had colorectal cancer), and have some indicator for being high risk (i.e., have a positive FOBT, first degree family member, history of rectal bleeding, or tubular adenoma on prior colonoscopy). More than just making colonoscopies affordable, the program eased access by allowing EFHC to directly schedule patients into dedicated appointment slots, thereby reducing the scheduling complexity for the patient. Further, pre-procedure preparation instructions and materials were given at EFHC to reduce literacy barriers and increase compliance. Patients for whom transportation was a barrier were given the option of being chaperoned to and from the appointment by an EFHC staff member.

#### Improvements in CRC screening rate

By October, 2009, the screening rate at EFHC had more than doubled to 43%. Because the changes above were implemented concurrently, it is not known which of these system changes was most responsible for the increase in the CRC screening rate. The multiple strategies employed at EFHC and the relatively high rate of CRC screening achieved by these interventions means that the “usual care” group in this study is a strong comparator.

## Methods

### Study design and waiver of informed consent

This study is a randomized controlled clinical trial with randomization at the level of either the patient or the household (if two or more patients in the same household were eligible for the study); the latter was done to ensure that patients living in the same household would not be randomized to different study arms, because the outreach to one would likely effect the outcome of the other. This study was approved by the Northwestern University Institutional Review Board (IRB). It was approved with a waiver of patient informed consent for randomization because it met previously established criteria: 1) very low risk to patients, as is typically the case for interventions designed to improve adherence to national guidelines, 2) informed consent could not be obtained without seriously threatening the validity of the study (i.e., if we attempted to obtain informed consent, patients in the usual care arm would be made aware of their need for repeat CRC screening), and 3) all patient information (e.g., demographic, diagnoses) and outcomes were obtained using data collected during the routine course of their care by querying the EHR database. We have used this same approach in prior comparative effectiveness studies, including a previous trial of outreach to improve CRC screening [14]. Conducting this study with a waiver of informed consent allows for the entire population of eligible patients to be included in the study, thereby achieving a true assessment of effectiveness without selection bias. After IRB approval, this study was also approved by EHFC’s Research Review Committee.

#### Inclusion and exclusion criteria

The eligibility criteria are shown in Table 1. The inclusion criteria were age 51–74 years, preferred language listed as English or Spanish, and an FOBT completed with negative result between March 7, 2011 and March 6, 2012. We excluded patients with any of the following: (1) colonoscopy within 10 years, (2) flexible sigmoidoscopy within 5 years, (3) medical conditions suggesting CRC screening through FOBT may be inappropriate, including chronic diarrhea, inflammatory bowel disease, iron deficiency, previous colonic polyp, use of medications in the previous 1 month that elevate the risk of a false-positive FOBT (i.e., plavix or warfarin), and medical conditions that make CRC screening inappropriate (metastatic cancer or previous total colectomy).

**Table 1:** Criteria for identifying patients eligible for the study

**Inclusion**	**Criteria**
Age	51-74 years at start of study
Language	English or Spanish
FOBT done in year before study	FOBT completed in year before study
	“Screening for colon cancer” (or related codes) entered as an encounter diagnosis in year before study
	FOBT result negative
**Exclusion**	
Screening by another test	Colonoscopy (CPT code 45.23) within 10 years
	Flexible sigmoidoscopy (CPT code 45.24) within 5 years
Conditions that suggest FOBT screening may be inappropriate	Chronic diarrhea (ICD-9 787.91)
	Inflammatory bowel disease: Crohn’s (CPT code 555.xx) or Ulcerative Colitis (CPT code 556.xx)
	Iron deficiency anemia (CPT code 280.9)
	Gastrointestinal bleeding (CPT code 578.xx)
	Colonic polyp (CPT code 211.3)
	Malignant neoplasm of colon and other specified sites of colon and large intestine (ICD-9-CM codes 153.X, 154.0, 154.1, 197.5, V10.05)
Conditions that make colorectal cancer screening inappropriate	Total colectomy (CPT codes 44150–44153, 44155–44156, 44210–44212; ICD-9-CM codes 45.8)
Medications that may affect the decision to screen or use FOBT	Clopidogrel, warfarin, or dabigatran

#### Identification of eligible patients and randomization

We used structured query language (SQL) to analyze EFHC’s EHR data to identify eligible patients. The accuracy of the query was confirmed by reviewing 25 random patients identified as being eligible and overdue and 25 identified as not being overdue. The query was also used to obtain patients’ medical record numbers, demographics, preferred language, number of chronic medical conditions, number of visits to the clinic in the past 12 months, primary care provider, and contact information.

To prevent having patients in the same household being assigned to different groups, we first identified all eligible patients who had the same address (28 patients, 14 households). We then used a random number generator to assign households in the first half of the list to the intervention (N = 7) and the remainder to the usual care group (N = 7). This process was repeated for all remaining eligible patients (N = 422) with a unique address.

### Conceptual framework

Most quality of care problems are due to a combination of patient, provider, and system-level factors. Any one of these may be rate-limiting so that care remains suboptimal even if most other aspects of care are done well. For example, if the care team routinely recognizes when patients are due for CRC screening but the patient does not understand why s/he needs to be screened or the instructions are intimidating, the test may not be performed. For this study, we developed a conceptual framework of likely barriers to optimal care and then designed the multifaceted intervention to address each of these barriers (Table 2). We believe that the components of the intervention may each have small, incremental effects that are both additive and synergistic, although we will not be able to determine the incremental effects of all individual components (see analysis plan below).

**Table 2:** Conceptual framework for the intervention components

**Barrier**	**Intervention component(s) to address barrier**
No clinical systems in place to identify patients who need repeat screening, unless they present for care	EHR query to identify patients due for repeat screening; outreach to patients (half randomized to intervention)
No personal systems in place for patients to track when preventive services are due	Automatic phone/text reminders to remind patients they are due for screening
Low adherence to repeat screening because of financial and/or logistical barriers	Mail FOBT kits to patients
Patients forget to return FOBT	Automatic phone/text reminders to patients who do not return FOBT within 2 weeks
Low priority and/or risk perception for CRC	Call from CRC Screening Coordinator at 3 months to patients who do not complete FOBT to explain need for screening
Change of phone number and/or address makes initial reminders unsuccessful	When CRC Screening Coordinator calls at 3 months, he can use updated information (i.e., from a recent visit)
Patients do not understand instructions	Mailed FOBT kits include plain language information, instructions, and direct phone number for CRC Screening Coordinator
Lack of understanding of polyps, CRC, and recommendation for FOBT screening	Call from CRC Screening Coordinator at 3 months to patients who do not complete FOBT to explain why they need repeat screening, answer questions, and mail another FOBT if requested; letter from CRC Screening Coordinator when FOBT results are negative to remind patients to repeat screening in 1–2 years and give due date

### Development of outreach tools

#### Letters, automated telephone calls, and texts

We developed simple, short messages to remind patients they are due for screening. All calls, text, and print materials were developed in English and translated into Spanish. Bilingual staff worked to ensure equivalency of the two language versions. EFHC’s EHR includes patients’ preferred language, and all messages are first sent in the preferred language; patients are told that materials are also available in the other language from the CRC Screening Coordinator; this strategy will address problems if the language preference recorded in the EHR is incorrect. After initial versions were developed, we held a focus group and had EFHC patients with Spanish listed as their preferred language review, critique, and provide feedback on all outreach materials. The final versions of the letter, text message, and automated phone call message are shown in the Additional file 1.

#### Development of fecal occult blood testing kit instructions

For this project, we are using the PolyMedco OC-Light Fecal Occult Blood Test, an immunoassay that detects human hemoglobin in feces [15]. This class of screening test is referred to as fecal immunochemical tests (FIT). It can detect hemoglobin in concentrations as low as 0.05 micrograms/mL. It does not cross-react with animal hemoglobin from consumed meat, and it does not cross-react with vegetables containing peroxidase (e.g., horseradish, cauliflower, broccoli) or toilet bowl cleaners. The reported sensitivity and specificity of the OC Light for detecting CRC are 91.0% and 93.8% [16], which is consistent with the reported range for other FIT tests [1]. It is a single-sample test, which decreases the demand on patients and increases adherence compared to guaiac cards that require multiple samples [17-20]. This FIT test was used for both the intervention and usual care groups; all EFHC clinics were stocked with the FIT test and staff was trained in the use of this test so it became the standard of care for CRC screening.

The manufacturer’s instructions for the test were not designed for a patient population with low literacy. We therefore undertook a comprehensive redesign of the instructions, including identification of each key step, development of short instructions for each step, and creation of graphics to clarify and reinforce the text. The instructions were first reviewed by a focus group of EFHC staff. After revisions, we conducted a focus group and asked patients to simulate using the test and follow the instructions without any additional preparatory information. Multiple revisions were made in response to patient and staff suggestions and incorporated into final version of instructions. The English and Spanish versions of FIT instructions are shown in the Additional file 1.

### Two-phase intervention

#### Initial intervention

We group patients into weekly cohorts based on the date of their eligible FOBT between March 2011 and March 2012. Every Monday, all patients due for repeat FOBT screening in the coming week have a FIT kit mailed to their home, along with a letter from their primary care provider and the FIT instructions. We then send the automated phone call and two days later a text message via the commercial system (CallPointe; Tuscon, AZ) that EFHC uses routinely for patient reminders and health promotion messages. The system software allows us to track the number of completed phone and text messages, and whether the phone message is answered by a person or a machine.

Two weeks after the initial reminders and FIT kit mailing, we query the EHR to identify patients who did not return the mailed kit. Those who do not return the kit receive another automated phone call and text message.

#### Personal calls by CRC Screening Coordinator at three months

Three months after the initial outreach and FIT kit mailing, we again query the EHR to identify patients in the intervention group who have not completed screening; the CRC Screening Coordinator then attempts to contact them by phone. We chose this three month cutoff because in previous studies it took 3–4 months to see the full effect of an initial outreach intervention [14,21]. By waiting three months until we can see the full effect of the initial intervention, we should be able to estimate the incremental effectiveness of the CRC Screening Coordinator calls beyond the effects of the initial outreach. In addition, by waiting three months to make personal calls, we may be able to contact patients who have changed phones or addresses but who had a clinical encounter during this period and now have updated information in the EHR. If the CRC Screening Coordinator is able to speak with the patient, he explains why repeat testing is needed, answers questions, and explores barriers to completing the FIT. If the patient verbalizes willingness to complete the test, another FIT kit is mailed to them. Persuasion theory suggests that obtaining verbal commitment will increase the proportion of patients who will actually complete the test [22,23]. If the CRC Screening Coordinator only obtains voicemail, a standard script is read reviewing key points and leaving a direct telephone number for contact.

### Education tools for patients after FIT testing

#### Follow-up of negative screening tests

FIT kits are processed by the EFHC laboratory and results entered into their EHR. FIT results are sent to the CRC Screening Coordinator. If the FIT is negative, the Coordinator notifies the patient by mail; the letter (Additional file 1) includes a reminder that the test must be repeated in one year along with a reminder card. Our goal is to prime patients for the second round of reminders/mailings they will receive in one year and to encourage them to initiate contact on their own. This priming may be important because many patients may change phone numbers, addresses, or providers over the course of a year.

#### Follow-up of positive screening tests

Nationally, a high proportion of patients with a positive FOBT do not complete a diagnostic evaluation [24-32]. To address this, if the FIT is positive (i.e., blood detected), the CRC Screening Coordinator works with the patient’s physician to arrange follow-up colonoscopy. Patients are tracked and reminded until this is completed or until the patient refuses. The date of completed colonoscopy, findings, pathology results, and recommended date for repeat screening (if completely normal) or surveillance (if abnormal) are added to the patient’s problem list in the EHR.

During the first few months of the project, we learned that patients often struggled to understand the colonoscopy preparation instructions, which had high literacy and cognitive demands. This is consistent with previous research showing that colonoscopy preparation is suboptimal, which decreases the sensitivity of the test for finding polyps and may even require repeat colonoscopy [33,34]. Therefore, we applied the same methods described above for developing the FIT instructions to create simplified colonoscopy preparation instructions (see Additional file 1). We are also working to develop a video that explains to patients what a colonoscopy is and how it is performed to ease their concerns and maximize completion rates. Previous studies have reported that only 25-59% of patients with positive FOBTs undergo diagnostic colonoscopy [24,25,27-31,35-38]. Although our study was not designed to test whether the tools we developed will improve completion rates, we hope that this may be possible in future studies.

### Outcome assessment

The primary outcome for the study is completion of a FOBT within six months of the date when a patient first became due for repeat screening (i.e., one year after their qualifying FOBT). The outcome is assessed by querying the EHR for both intervention and usual care patients to determine if a FOBT screening test was completed. By relying only on the EHR for outcome assessment for both the intervention and usual care group, we hope to avoid any bias that might occur in the rates of detection of FOBT completion. Specifically, if the CRC Screening Coordinator was aware that a FIT was performed but the results were not documented in the EHR, the patient would still be classified as having not completed screening. Using only EHR data for outcome assessment is conceptually similar to blinded outcome assessment.

We have found that some patients randomized to the intervention have a repeat FOBT done at a clinical encounter shortly before the date that they are due for the intervention. The intervention is not conducted for these patients. Equal numbers of patients in the control group are expected to complete repeat testing prior to their due date, but we remain blinded to the outcomes in the control group. Because these patients were randomized prior to knowing their outcomes, we will include them in the main analyses.

### Power calculation

The repeat screening rate for patients at EFHC was not known at the time this study was designed because many eligible patients had only recently completed their first FOBT. We assumed that 35% of patients in the usual care group will achieve the primary outcome, similar to the rate in previously published studies. To detect a 10% difference (45% vs. 35%) with 80% power (2-tailed alpha = .05), we would need 752 patients (376 in each arm). This is less than the 800 patients that we estimated will be eligible for the study. We think intervention effects less than 10% are not important to detect because it would not be substantial enough to justify the efforts required to implement this type of an intervention in CHCs.

### Analytic plan

The rate of FOBT completion for the intervention and usual care groups will be analyzed with an intention to treat approach that includes all patients randomized; this will provide a true picture of the effectiveness of the intervention that will allow other health care providers (especially similar CHCs) to know the probable benefits of implementing this intervention across their health care center or system.

In addition, we will conduct a subgroup analysis stratified by the number of visits to the clinic during the follow up period to see if the intervention’s main benefit is to obtain CRC screening for patients with infrequent clinic visits. We will also examine the fidelity of the intervention, including the proportion of patients who did not receive the mailed FIT kit (i.e., returned to sender because of incorrect address), the proportion who had the text message completed, the proportion that had the automated call completed, and the proportion of automated calls answered a) in person or b) by an answering machine. We will conduct logistic regression to analyze the association between the components of the intervention that were received (both the number and type) and the likelihood of FIT completion. We will also assess the incremental value of the three-month outreach calls by the CRC Screening Coordinator.

## Discussion

This study will determine whether a multifaceted intervention is able to improve rates of repeat FOBT for CRC screening compared to usual care. It is important to stress that “usual care” at EFHC is at a high standard, consisting of team-based care for promoting CRC screening, point-of-care alerts for clinicians, and performance measurement and feedback to providers. These efforts to improve CRC screening are probably far greater than those in most private practices and CHCs. Health services research studies sometimes have relatively weak “usual care” groups; although the care for the control group may be “typical” care, it is not consistent with what the literature has shown to be evidence-based best practice. Thus, it can be hard to determine whether a new intervention truly has a marginal value beyond what has already been shown to be beneficial. In contrast, the usual care group in this study includes multiple evidence-based strategies to improve preventive service delivery through point-of-care quality improvement methods.

This study will examine use of an EHR system for population-based disease management and outreach among vulnerable patients cared for in CHCs. Very few studies have examined the benefit of using EHR-based interventions in this setting. These data are important, as more CHCs are seeking to become patient-centered medical homes with good health information technology (HIT) infrastructure. In addition, our study design will allow us to separate the effects of low cost technology (automated reminders) followed by more costly efforts (telephone outreach by a CRC Screening Coordinator). This tiered outreach strategy could help clinics maximize efficiency and make decisions as to when it is helpful to use more resource intensive strategies.

Although the main goal of this study is to determine the comparative effectiveness of the intervention compared to usual care, we also want to see if it is possible to achieve the high rates of adherence to CRC screening with annual FOBT that are necessary to achieve measurable reductions in CRC mortality. Few studies have examined the rate of repeat FOBT testing [11,12,39], and to our knowledge none have been conducted in populations with a high prevalence of barriers to screening (e.g., low literacy, varied cultural norms, transportation difficulties). A study from the Department of Veterans’ Affairs found that only 42% of men received adequate screening (FOBTs in at least 4 out of 5 years) [12]. A study of managed care plan enrollees in Washington state found that only 44% completed repeat screening within 2 years [11]. The assumption that FOBT is an effective CRC screening strategy presumes it will be done at least biennially, and cost-effectiveness studies of CRC screening strategies have found that the results are sensitive to the rate of adherence [13]. Our study will provide critical information for providers and policymakers as they consider different strategies (i.e., FOBT, flexible sigmoidoscopy, colonoscopy) to increase CRC screening among vulnerable populations.

If effective, the study intervention will serve as a model for CHCs of how to improve rates of repeat FOBT among vulnerable populations. Our intervention relies on technology that is increasingly available and declining in cost, including EHR systems, automated phone and text messaging, and FITs for CRC screening. This approach was taken to try to ensure generalizability and allow us to rapidly disseminate the intervention through networks of CHCs.

Conversely, if the intervention is not effective or if rates are suboptimal despite the intervention, our findings will spur future research and policy analyses about alternative screening strategies to address disparities. Endoscopy is more sensitive than FOBT for CRC screening and needs to be conducted less often. However, the costs of endoscopy are higher and many CHCs have limited access to endoscopy. Although there are significant barriers to using endoscopy for CRC screening, previous studies have shown that nurses can be trained to do flexible sigmoidoscopy effectively and safely [40-43]. Endoscopy is an acceptable screening strategy among populations with CRC screening disparities [44-50]. A study of screening colonoscopy for patients in East Harlem (55% Hispanic, 33% African American) found that 66% of patients completed colonoscopy with the aid of a patient navigator [51]. In addition, other studies have found that many patients cared for in safety net settings would prefer endoscopy to FOBT [47,50,52]. Such screening alternatives may need increased scrutiny should our intervention fail to achieve high rates of annual screening.

## Competing interests

The authors declare that they have no competing interests.

## Authors’ contributions

Obtaining funding: DWB, KC, MW. Study design: DWB, DB, KC, MW. Development of study intervention components: all. Writing the manuscript: DWB, TB, DB, JW, SG. Critical revision of the manuscript and final approval: all.

## Pre-publication history

The pre-publication history for this paper can be accessed here:

http://www.biomedcentral.com/1472-6963/13/153/prepub

## Supplementary Material

Additional file 1 Improving Rates of Repeat Colorectal Cancer Screening Appendix. Click here for file
