# Supplementary material for: Design of a randomized controlled trial to assess the comparative effectiveness of a multifaceted intervention to improve adherence to colorectal cancer screening among patients cared for in a community health center
Source: BMC Health Serv Res. 2013 Apr 29;13:153. doi: 10.1186/1472-6963-13-153 (PMC3656775; doi:10.1186/1472-6963-13-153)
Supplement: Additional file 1 — Improving Rates of Repeat Colorectal Cancer Screening Appendix. [file 1472-6963-13-153-S1.pdf]

Center for ADVANCING EQUITY in Clinical Preventive Services

Improving Rates of Repeat Colorectal Cancer Screening

Appendix

Northwestern University, Feinberg School of Medicine

Contents

Patient Letter Included with Mailed FIT 3

Automated Phone Call 4

Automated Text Message 5

Results Notification Letter Sent to Patients with Negative FIT 6

CRC Screening Reminder Card 7

FIT Instructions 8

Colonoscopy Prep Instructions 10

Patient Letter Included with Mailed FIT

**ENGLISH**	Dear XXX,<br><br>A year ago, you did a test to check for colon cancer. Your test was normal.<br>But, colon cancer can start any time. And when cancer is starting, you do not feel anything. To protect yourself from colon cancer, you need to do this test every year. It is time to do the test again.<br>Last time, you put some stool (poop) on three cards. The test checked for hidden blood in your stool, which is a sign of colon cancer. The test is much easier this year. You just need to collect stool one time, and you can eat whatever food you usually eat before the test. We have sent you the test kit with this letter. Just follow the instructions. Mail it back to us as soon as you have done the test. The test and the postage are free.<br><br>This simple test could save your life. Do it and send it in right away!<br><br>If you have any questions about the test or how to do the test, please call XXX, the colon cancer screening coordinator, at xxx-xxx-xxxx.<br><br>Sincerely,
**SPANISH**	Estimado Don XXX, {or} Estimada Doña XXX,<br><br>Hace un año atrás usted se hizo una prueba para detectar cáncer de colon. Esa <u>prueba</u> salió normal.<br><br>Pero el cáncer de colon puede comenzar en cualquier momento. Y cuando el cáncer está empezando, uno no siente <u>nada</u>. Para protegerse del cáncer de colon, usted debe hacerse esta prueba cada año. Ahora le toca hacerse la prueba nuevamente.<br><br>La última vez, usted puso un poco de materia fecal (<u>caca</u>) en tres tarjetas. La prueba buscó la presencia de sangre oculta en su materia fecal (<u>caca</u>), lo cual es una señal de cáncer de colon. Este año la prueba es mucho más fácil. Sólo tiene que recolectar su materia fecal (<u>caca</u>) una vez, y puede comer cualquier comida que usted por lo general come antes de la prueba.<br><br>Le hemos enviado con esta carta el material para hacer la prueba. Simplemente siga las instrucciones. Envíenosla de regreso apenas haya hecho la prueba. La prueba y el costo de mandarla por correo son gratuitos para usted.<br><br>Esta sencilla prueba podría salvarle la vida. ¡Hágala y envíenos su prueba de inmediato!<br><br>Si tiene alguna pregunta sobre la prueba o sobre cómo se hace, por favor llame a XXX, coordinador bilingüe de pruebas de cáncer de colon, al teléfono xxx-xxx-xxxx.<br><br>Atentamente,

Automated Phone Call

	INITIAL	REMINDER (2 WEEKS)*
**ENGLISH**	I'm calling for <FULL NAME> from Erie Family Health. It's time for you to get tested again for colon cancer. A year ago, <FIRST NAME> did a test for colon cancer that was normal. But colon cancer can start at any time. So, to protect yourself from colon cancer, you need to do this test every year. To make it easier for you, **we are mailing you the test**. Do the test as soon as **you get it**, and mail it in right away! It's easy, and it's free! This simple test could save your life	I'm calling from Erie Family Health **with a reminder** for <FULL NAME>. It's time for you to get tested again for colon cancer. A year ago, <FIRST NAME> did a test for colon cancer that was normal. But colon cancer can start at any time. So, to protect yourself from colon cancer, you need to do this test every year. To make it easier for you, **we mailed you the test recently**. Do the test as soon as **possible**, and mail it back right away! It's easy, and it's free! This simple test could save your life**. If you have questions about the test, please call XXX, the colon cancer screening coordinator, at xxx-xxx-xxxx.**
**SPANISH**	Estoy llamando del Centro de Salud Erie para hablar con <FULL NAME>. Le toca hacerse la prueba de cáncer de colon de nuevo. Hace un año atrás, <FIRST NAME> se hizo una prueba de cáncer de colon y los resultados salieron normales. Pero el cáncer de colon puede empezar en cualquier momento. Por lo tanto, para protegerse del cáncer de colon, debe hacerse la prueba cada año. Para que le resulte más fácil, **le vamos a mandar** por correo la prueba. **Hágala apenas la reciba** y envíela por correo de inmediato. Es fácil de hacer, y es gratis. Esta sencilla prueba puede salvarle la vida.	Estoy llamando del Centro de Salud Erie para hablar con <FULL NAME>. Le toca hacerse la prueba de cáncer de colon de nuevo. Hace un año atrás, <FIRST NAME> se hizo una prueba de cáncer de colon y los resultados salieron normales. Pero el cáncer de colon puede empezar en cualquier momento. Por lo tanto, para protegerse del cáncer de colon, debe hacerse la prueba cada año. Para que le resulte más fácil, **hace dos semanas le enviamos** la prueba por correo. **Hágala lo más pronto posible** y envíela por correo de inmediato. Es fácil de hacer, y es gratis. Esta sencilla prueba puede salvarle la vida. **Si tiene alguna pregunta sobre la prueba o sobre cómo se hace, por favor llame a XXX, coordinador bilingüe de pruebas de cáncer de colon, al teléfono xxx-xxx-xxxx.**

*Differences between initial and reminder scripts in bold.

Automated Text Message

	Initial	Reminder (2 weeks)
**English**	Erie Family Health: Time to repeat colon cancer test. Cancer can start any time. Do test every year. You **will receive FREE** test in the mail **this** week.	Erie Family Health: Time to repeat colon cancer test. Cancer can start any time. Do test every year. You **should have received** test in mail **last** week.
**Spanish**	Centro de Salud Erie: Le toca repetir la prueba de cáncer de colon. Ud. debe hacer la prueba cada año. **Recibirá la prueba GRATIS** por correo **esta** semana.	Centro de Salud Erie: Le toca repetir la prueba de cáncer de colon. Ud. debe hacer la prueba cada año. **Debería haberla recibido** por correo **la** semana **pasada**.

Results Notification Letter Sent to Patients with Negative FIT

**English**	Dear XXX<br><br>Good news! The test for colon cancer that you sent in was normal. There was no blood.<br><br>Just remember that to protect yourself you need to do this test every year. So write down that you should have this test done again in one year. We have included a reminder card with the date when you should have this done again.<br><br>Now that you have been tested for colon cancer, help protect your family and friends! Everyone between the ages of 50 and 75 should be tested. Tell them how easy it is and encourage them to get tested.<br><br>Sincerely,
**Spanish**	Estimado Don XXX, {or} Estimada Doña XXX,<br><br>¡Buenas noticias! La prueba del cáncer de colon que usted nos envió dio un resultado normal. No había sangre.<br><br>Sólo recuerde que para protegerse tiene que hacerse esta prueba cada año. Por lo tanto anote que debe hacerse esta prueba nuevamente en un año. Hemos incluido una tarjeta como recordatorio, con la fecha en que debe hacérsela nuevamente.<br><br>Ahora que ya se ha hecho la prueba del cáncer de colon, ayude a proteger a su familia y a sus amigos. Todas las personas entre las edades de 50 y 75 años deberían hacerse la prueba. Dígales lo fácil que es y anímelos a hacerse la prueba.<br><br>Atentamente,

CRC Screening Reminder Card

ENGLISH	**Protect Yourself from Colon Cancer.**<br>**Get Checked Every Year!**<br>Your last test was normal. It was done on<br>____________________<br>You should have this test done again on<br>____________________<br>Talk with your doctor, or call Erie Family Health Center at xxx-xxx-xxxx and we will mail you a free test kit.
SPANISH	**Protéjase Contra Cáncer del Colon.**<br>**¡Repita Su Chequeo Anualmente!**<br>Su último chequeo salió normal. Lo hizo el<br>____________________<br>Le toca hacerlo de nuevo el<br>____________________<br>Hable con su médico o llame al Centro de Salud Erie al xxx-xxx-xxxx, y le enviaremos el equipo gratuito.

How to use the test

Read all these instructions before doing the test!

1. Unfold the paper and put it in your toilet bowl, on top of the water.

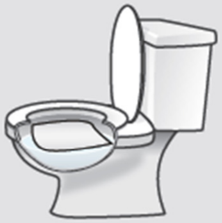

2. Have a small bowel movement on top of the paper. Collect the stool (poop) right away! Do not let the paper sink or let the stool get wet.

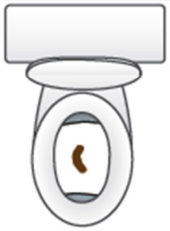

3. Get the test bottle. Check to make sure it has your name and birth date on it. Unscrew the green cap. Pull the cap straight out.

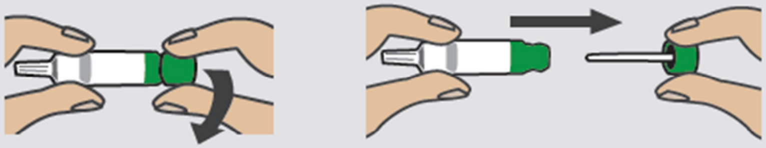

4. Scrape the stool (poop) with the stick. Scrape it a few times until the grooves near the end of the stick are filled completely with stool.

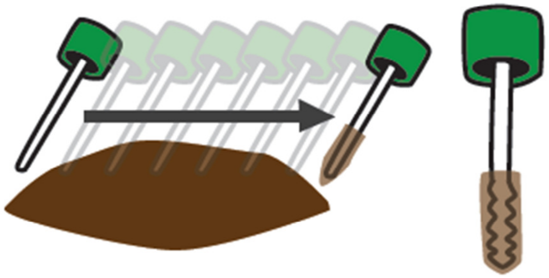

5. Push the stick straight into the test bottle. Press in hard, and screw the cap on tightly. Wipe off the outside of the bottle with some tissue. Flush the toilet. It is safe to flush the paper.

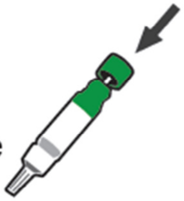

6. Wrap the bottle with the small pad.

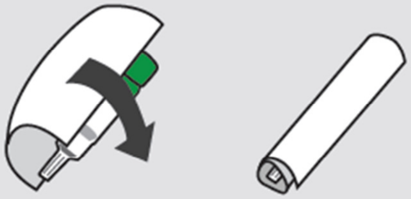

7. Put the bottle and the pad inside the plastic bag. Put the plastic bag in the return envelope.

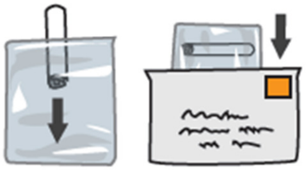

8. Seal the envelope. Write today's date on the envelope.

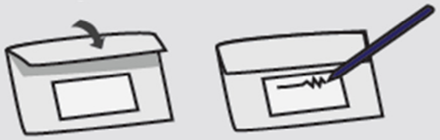

9. Mail the test to us right away. Now you are done!

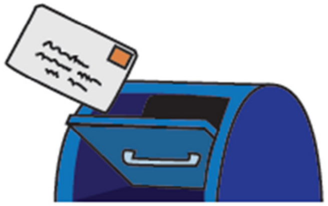

Questions? Call us at **xxx-xxx-xxxx**

Cómo usar la prueba ***¡Lea todas las instrucciones antes de hacer la prueba!***

1. Desdoble el papel y póngalo adentro de la taza de baño, sobre el agua.

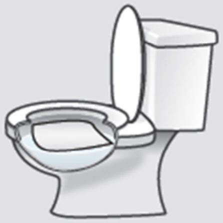

2. Haga una cantidad pequeña de excremento (caca) sobre el papel. ¡Recolecte la materia fecal (caca) de inmediato! No deje que el papel se hunda o deje que la materia fecal (caca) se moje.

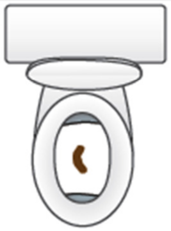

3. Agarre el frasquito de la prueba. Fíjese que tenga su nombre y su fecha de nacimiento. De le vuelta a la tapita verde para destaparla, y jálela para afuera.

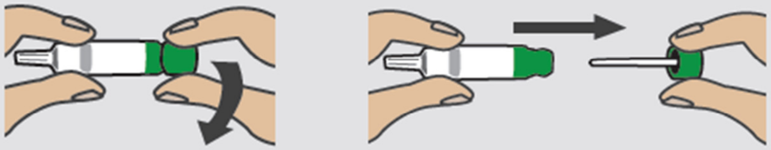

4. Con el palito, raspe la materia fecal (caca). Raspe unas cuantas veces hasta que los espacios pequeños cerca del final del palito estén totalmente cubiertos de materia fecal (caca).

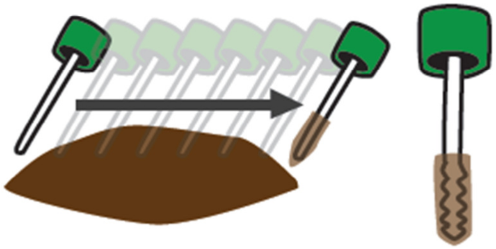

5. Meta el palito derecho hacia adentro del frasquito de la prueba. Empújelo fuerte para que entre, y ponga la tapa y ciérrela bien. Con un poco de papel higiénico, limpie el exterior del frasquito. Haga correr el agua de la taza de baño. No hay problema en echar el papel a la taza de baño.

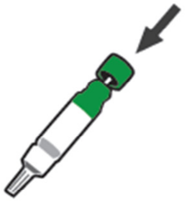

6. Envuelva el frasquito con la almohadilla.

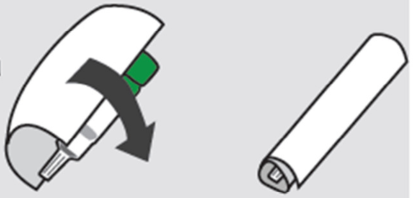

7. Ponga el frasquito y la almohadilla dentro de la bolsita de plástico. Ponga la bolsita de plástico en el sobre para enviar la prueba.

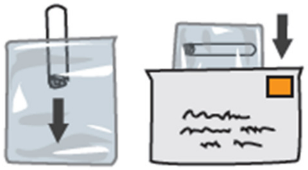

8. Cierre bien el sobre. En el sobre, escriba la fecha de hoy.

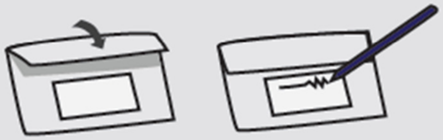

9. Envíenos de inmediato la prueba. ¡Listo, ya terminó!

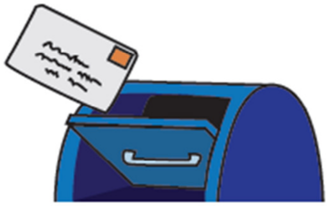

¿Alguna pregunta? Llame a **xxx-xxx-xxxx**

Instructions for your Colonoscopy

Read all these instructions as soon as you get them.

Your colonoscopy is scheduled for:

____

You need to arrive at:

____

It usually takes about:

____

Take these medicines on the day of your colonoscopy:

____

Other instructions:

____

Questions? You can call ________________ or any other nurse at ________________ by calling xxx-xxx-xxxx

nurse site

Getting ready for your test

About your test

- Your doctor has ordered a colonoscopy. For this test, the doctor puts a thin tube inside your colon. It looks for cancer or polyps. Polyps are growths that can turn into cancer. If a polyp or an early cancer is found, your doctor can take it out.
- It is important that your colon is cleaned out and empty before the test. You will drink a special mix to make all the stool (poop) come out. This will let your doctor see if anything is wrong.
- You will be given medicine during the test to make you feel comfortable and sleepy. You will not feel any pain at all.

Preparing for your test

- Plan to take off work the day of the test. You will be sleepy after the test and not able to work. You can go to work the next day.
- You must have someone take you home after the test. Even if you are taking a cab or bus, make sure someone can help you get home safely.
- Buy plenty of clear liquids: water, yellow Gatorade, Sprite, apple juice, or chicken broth without noodles.

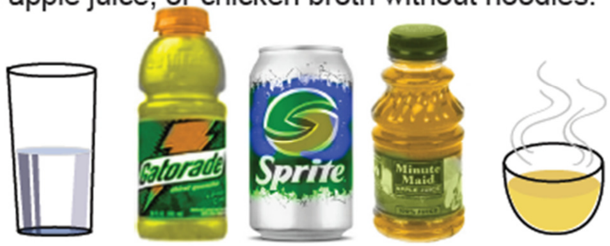

The day before your test

- Do not eat any food. You may eat after the test.
- Drink plenty of clear liquids all day long: water, yellow Gatorade, Sprite, apple juice, or chicken broth without noodles.

Questions? You can call _______________ or any other nurse at _______________ by calling XXX-XXX-XXXX

nurse site

The night before the test ____________________
mm/dd/yy

- Follow these steps beginning at ________PM
- Plan to be home near a toilet, because the mix will make you go to the bathroom very often.

1. Open the MoviPrep box.
It has 2 packet As, 2 packet Bs, and a plastic jar.

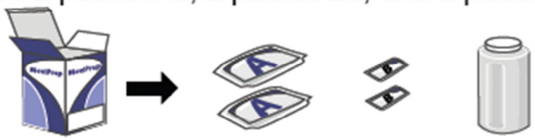

2. Empty 1 packet A and 1 packet B into the jar.

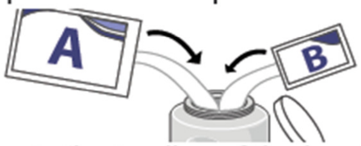

3. Add water to the top line of the jar. Mix well. Do <u>not</u> add ice.

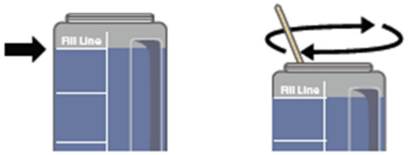

4. The jar has four lines. Every 15 minutes, drink the mix to the next line (about 1 cup), until the jar is empty.

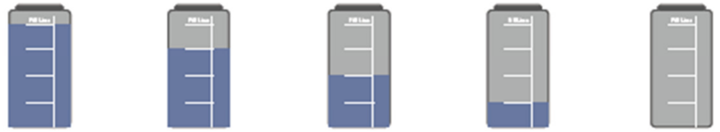

____:00 PM ____:15 PM ____:30 PM ____:45 PM ____:00 PM

5. Drink 2 cups of one of these liquids:
water, yellow Gatorade, Sprite, apple juice, or chicken broth without noodles.

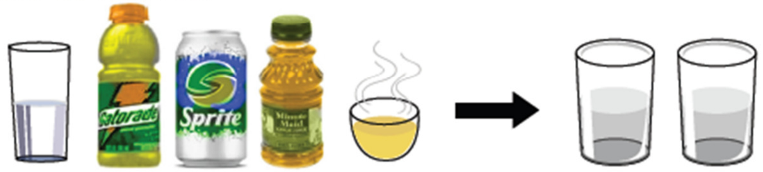

The morning of the test ____________________
mm/dd/yy

- Follow these steps beginning at ________________AM
- You will repeat the same steps you did last night.

1. Do not eat any food until after the test.

Take your medicines listed on the first page of these instructions.

2. Empty 1 packet A and 1 packet B into the jar.

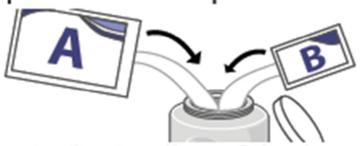

3. Add water to the top line of the jar. Mix well. Do <u>not</u> add ice.

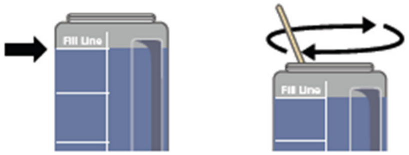

4. The jar has four lines. Every 15 minutes, drink the mix to the next line (about 1 cup), until the jar is empty.

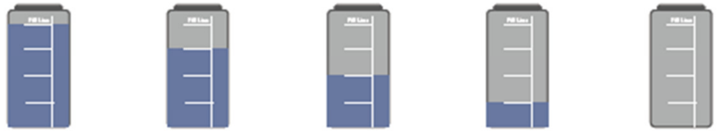

____:00 AM ____:15 AM ____:30 AM ____:45 AM ____:00 AM

5. Drink 2 cups of one of these liquids:
water, yellow Gatorade, Sprite, apple juice, or chicken broth without noodles.

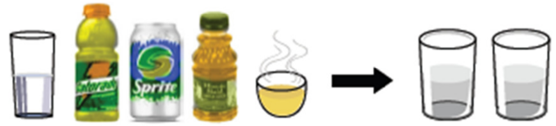

6. **Do <u>not</u> drink anything for 5 hours before your test.**

Tips for drinking the mix

- If you start feeling sick from the mix, try drinking it more slowly. Drink a smaller amount every 20 minutes until the jar is empty.
- If you prefer to drink the mix cold, put it in the refrigerator the night before to cool down. Do not add ice!

Checklist

When you are scheduling your colonoscopy:

- ☐ Schedule the day off from work
- ☐ Arrange for a ride home after the test. Someone must pick you up.
- ☐ Pick up MoviPrep box

1 week before your colonoscopy:

- ☐ Call your ride for the day of the colonoscopy
- ☐ Buy enough liquid (water, yellow Gatorade, Sprite, apple juice, or chicken broth without noodles) to drink
- ☐ Ask your doctor about changes to your medicines

The day before your colonoscopy:

- ☐ Do not eat food
- ☐ Drink liquid throughout the day (water, yellow Gatorade, Sprite, apple juice, or chicken broth without noodles)

The night before your colonoscopy:

- ☐ Make mix
- ☐ Drink mix
- ☐ Drink 2 cups of liquid (water, yellow Gatorade, Sprite, apple juice, or chicken broth without noodles)

The morning of your colonoscopy:

- ☐ Call your ride to remind them of the colonoscopy
- ☐ Make mix
- ☐ Drink mix
- ☐ Drink 2 cups of liquid (water, yellow Gatorade, Sprite, apple juice, or chicken broth without noodles)
- ☐ Do not eat or drink anything after this
- ☐ Get to appointment on time

Questions? You can call _______________ or any other nurse at _______________ by calling XXX-XXX-XXXX

nurse site

Instrucciones para su Colonoscopia

Lea todas estas instrucciones tan pronto las reciba.

Se ha programado su colonoscopia para:

____

Usted necesita llegar a las:

____

Por lo general la colonoscopia toma aproximadamente:

____

Tome estos medicamentos en el día de su colonoscopia:

____

Otras instrucciones:

____

¿Preguntas? Puede hablar con _______________ o cualquier otra enfermera en _______________ por llamar al XXX-XXX-XXXX

enfermera lugar

Preparándose para la prueba

Acerca de la prueba

- Su médico ha ordenado una colonoscopia. Para realizar esta prueba, el médico colocará un tubo delgado dentro de su colon. Buscará cáncer o pólipos. Los pólipos son tumores que pueden convertirse en cáncer. Si encuentra un pólipo o un cáncer temprano, su médico lo puede extirpar.

- Es importante que el colon esté limpio y vacío antes de la prueba. Usted beberá una mezcla especial para hacer que salgan todas las heces (caca). Esto permitirá que su médico vea si algo está mal.

- Se le dará un medicamento durante la prueba para que se sienta cómodo y tranquilo. Usted no sentirá ningún dolor en absoluto.

En preparación para la prueba

- Planee no ir a trabajar el día de la prueba. Usted estará soñoliento después de la prueba y no podrá trabajar. Podrá ir a trabajar al día siguiente.

- Deberá tener a alguien que lleve a casa después de la prueba. Aunque vaya a tomar un taxi o un autobús, asegúrese de que alguien pueda ayudarle a llegar a casa de manera segura.

- Compre muchos líquidos claros: agua, Gatorade amarillo, Sprite, jugo de manzana, o caldo de pollo sin fideos.

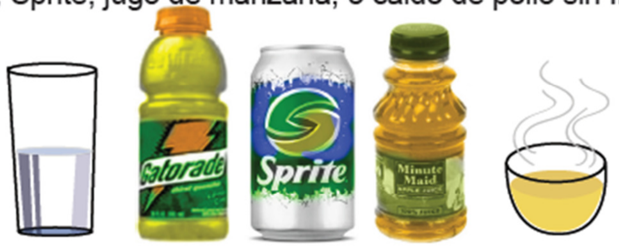

El día antes del examen

- No coma nada. Podrá comer después de la prueba.

- Beba muchos líquidos claros durante todo el día: agua, Gatorade amarillo, Sprite, jugo de manzana, o caldo de pollo sin fideos.

¿Preguntas? Puede hablar con _______________o cualquier otra enfermera en ________________ por llamar al XXX-XXX-XXXX

enfermera lugar

La noche antes del examen ____________________
mm/dd/aa

- Siga estos pasos comenzando a las _______PM
- Planee estar en casa cerca de un inodoro porque la mezcla le hará ir al baño muy seguido.

1. Abra la caja de MoviPrep.
Encontrará 2 sobres A, 2 sobres B, y una jarra de plástico.

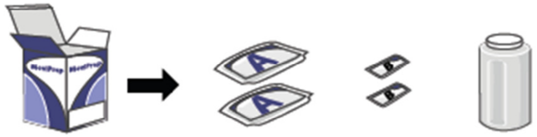

2. Vacíe 1 sobre A y 1 sobre B en la jarra.

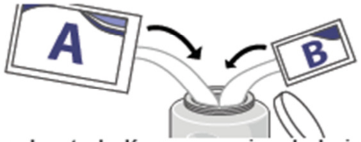

3. Añada agua hasta la línea superior de la jarra. Mezcle bien.
No agregue hielo.

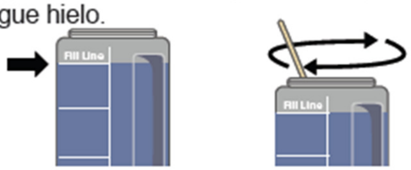

4. La jarra tiene cuatro líneas. Cada 15 minutos, beba la mezcla hasta la línea siguiente (aproximadamente 1 taza), hasta que la jarra esté vacía.

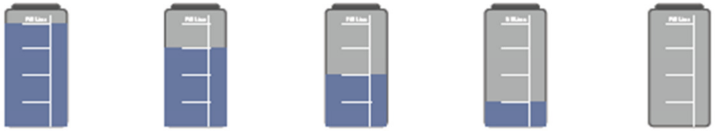

___:00 PM ___:15 PM ___:30 PM ___:45 PM ___:00 PM

5. Beba 2 tazas de uno de estos líquidos:
agua, Gatorade amarillo, Sprite, jugo de manzana, o caldo de pollo sin fideos.

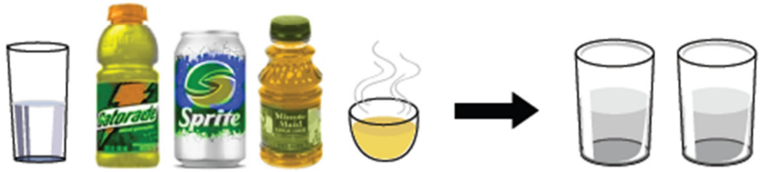

La mañana de la prueba ____________________
mm/dd/aa

- Siga estos pasos comenzando a las ________AM
- Repita los mismos pasos de la noche anterior.

1. No coma nada hasta después de la prueba.

Tome sus medicamentos enumerados en la primera página de estas instrucciones.

2. Vacíe 1 sobre A y 1 sobre B en la jarra.

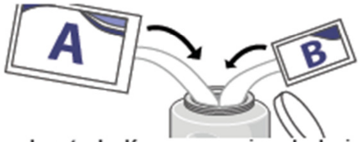

3. Añada agua hasta la línea superior de la jarra. Mezcle bien.
No agregue hielo.

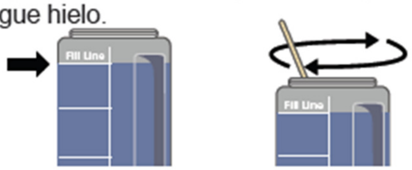

4. La jarra tiene cuatro líneas. Cada 15 minutos, beba la mezcla hasta la línea siguiente (aproximadamente 1 taza), hasta que la jarra esté vacía.

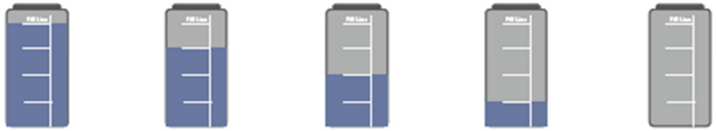

___:00 AM ___:15 AM ___:30 AM ___:45 AM ___:00 AM

5. Beba 2 tazas de uno de estos líquidos:
agua, Gatorade amarillo, Sprite, jugo de manzana, o caldo de pollo sin fideos.

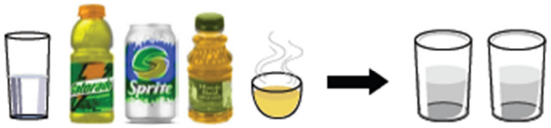

6. **No beba nada durante 5 horas antes del examen.**

Consejos para beber la mezcla

- Si comienza a sentirse mal al beber la mezcla, trate de beberla más lentamente. Beba una cantidad menor cada 20 minutos hasta que la jarra esté vacía.
- Si prefiere beber la mezcla fría, póngala en el refrigerador la noche anterior para que se enfríe. ¡No agregue hielo!

Lista de comprobación

Cuando esté programando su colonoscopia:

- ☐ Planee no ir a trabajar el día de la prueba
- ☐ Asegúrese de tener a alguien que le lleve a casa después de la prueba. Alguien debe recogerlo.
- ☐ Recoja la caja de MoviPrep

1 semana antes de su colonoscopia:

- ☐ Llame a la persona que le llevará a casa el día de la colonoscopia
- ☐ Compre suficientes líquidos para beber (agua, Gatorade amarillo, Sprite, jugo de manzana, o caldo de pollo sin fideos)
- ☐ Pregúntele a su médico acerca de cambios en sus medicamentos

El día antes de su colonoscopia:

- ☐ No coma nada
- ☐ Beba líquidos durante todo el día (agua, Gatorade amarillo, Sprite, jugo de manzana, o caldo de pollo sin fideos)

La noche antes de su colonoscopia:

- ☐ Prepare la mezcla
- ☐ Beba la mezcla
- ☐ Beba 2 tazas de líquidos (agua, Gatorade amarillo, Sprite, jugo de manzana, o caldo de pollo sin fideos)

La mañana de su colonoscopia:

- ☐ Llame a la persona que le ofrecerá transporte para recordarle la colonoscopia
- ☐ Prepare la mezcla
- ☐ Beba la mezcla
- ☐ Beba 2 tazas de líquidos (agua, Gatorade amarillo, Sprite, jugo de manzana, o caldo de pollo sin fideos)
- ☐ No coma ni beba nada después de esto
- ☐ Llegue a la cita a tiempo

¿Preguntas? Puede hablar con _______________ o cualquier otra enfermera en ________________ por llamar al XXX-XXX-XXXX

enfermera lugar
